# Researcher bias and the enduring gap between the world’s fastest men and women

**DOI:** 10.3389/fphys.2024.1360731

**Published:** 2024-03-06

**Authors:** Doug Rohrer

**Affiliations:** Department of Psychology, University of South Florida, Tampa, FL, United States

**Keywords:** running, world, record, men, women, progression, prediction

## Introduction

**FIGURE 1:**
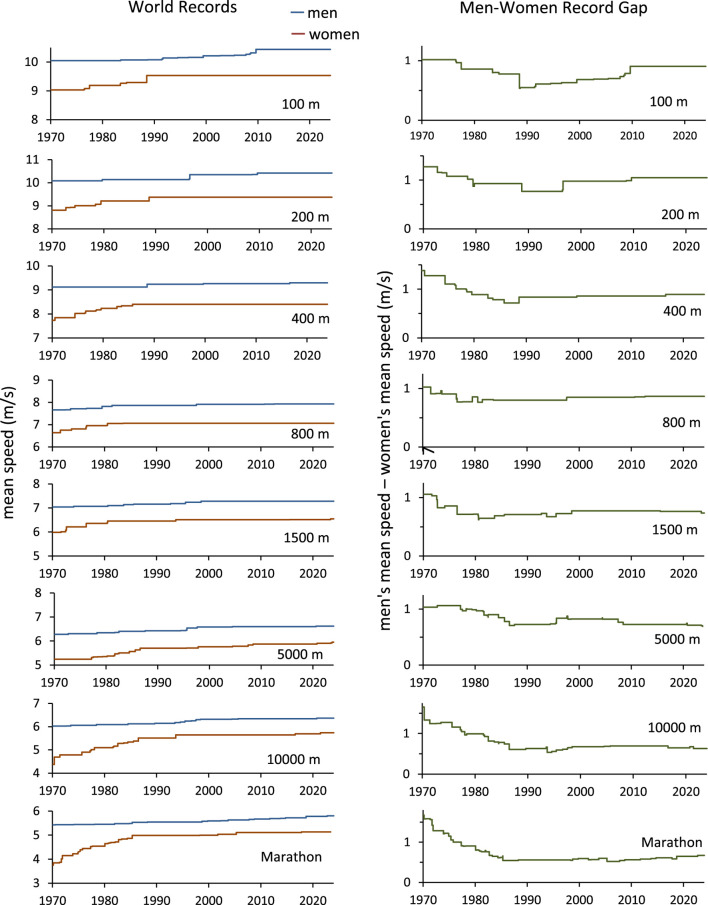
Progression of world records and the corresponding men-women record gaps. Mean speed = distance/time. Marathon = 42.195 km. Time span is January 1970 to January 2024.

Several researchers have argued that the gap between the fastest men and fastest women will narrow and then reverse. In an infamous 1992 letter to the journal *Nature*, two physiologists predicted that the world record sex gap in many events would mostly disappear before 2020 (Whipp and Ward, 1992). Since then, still other teams of researchers have asserted that the gap between the fastest men and women runners is vanishing, especially in long distance races (e.g., Bam et al., 1997; Tatem et al., 2004; Beneke et al., 2005; Le Mat et al., 2023). Yet the data plainly show that the men-women world record gaps essentially ceased narrowing in the 1980s (e.g., Holden, 2004; Cheuvront et al., 2005; Seiler et al., 2007; Thibault et al., 2010; Hallam and Amorim, 2022). In fact, in every Olympic running event from the 100-m dash to the 42.195-km marathon, the world record sex gap is about the same as it was in 1990^1^ (Figure 1).

This record gap does not mean that most men can outrun the fastest women. Indeed, the world’s fastest woman at any distance can outpace nearly all men. Still, the difference between the *fastest* men and the *fastest* women is large. In most Olympic track events, the world record for women is worse than the world record for 15-year-old boys.^2^

The large and enduring gap between the fastest men and women raises an obvious question: if the world record gap virtually ceased narrowing *before* 1990, why have so many arguments for reversing sex gaps appeared *after* 1990? The answer is that the published predictions of women outrunning elite men are flawed because of inappropriate research practices. These behaviors include cherry picking, invalid assumptions, misleading analyses, and the omission of contradictory evidence, all of which are illustrated below.

## Inappropriate research practices

Some predictions of women outperforming men are flawed because researchers ignored most of the data and instead focused on a small set of results consistent with their claim—a practice known as cherry picking. For instance, Tatem et al. (2004) predicted that the men-women gap in the 100-m dash will slowly narrow and eventually reverse, but the authors based their claim solely on the Olympic finals of the 100-m dash, which is a 10-s event that takes place only once every 4 years. Their analysis would have been far more reliable if they had drawn data from many competitions or instead examined the progression of world records, which can be set in any of the competitions sanctioned by the governing body of international track and field. It is also unclear why the authors focused solely on the 100-m dash. Other instances of cherry picking are better described as mere anecdotes. Beneke et al. (2005) argued that physiological sex differences favor women over men in ultramarathons, but they cited the outcomes of only two races, each won by a woman: the 2002 and 2003 iterations of a single event. Since then, men have won the event 19 of 20 times, usually by large margins.^3^

Other projections of reversing sex gaps have failed because researchers assumed that world records will improve at the same rate indefinitely, without ever reaching plateau. Whipp and Ward (1992) and Tatem et al. (2004) fit a line to the historical progression of world records, extended the line into the future, and predicted that the men-women record gap will reverse.^4^ The extrapolation of linear growth can produce absurd projections (e.g., Ellenberg, 2014, p. 32; Hays, 1994, p. 599), and the assumption of never-slowing growth is especially untenable when it underlies predictions of running records (Reinboud, 2004; Cheuvront et al., 2005; Seiler et al., 2007; Gelman and Nolan, 2017, p. 20). Moreover, the rate at which running world records improve began to flatten in the 1980s, years *before* Whipp and Ward and Tatem et al. published their projections (Figure 1). For example, the women’s marathon record improved rapidly during the 1970s and early 1980s, as women marathoners gained more opportunities to compete and receive compensation, but the progression slowed abruptly in the 1980s. Nevertheless, Whipp and Ward ignored this plateau, fit a line to the data, and predicted that the fastest women marathoners would surpass the fastest men in 1998.

Still other claims of women outrunning men are invalid interpretations of valid data. For instance, Le Mat et al. (2023) and Ronto (2023) compared the ultramarathon performances of men and women *finishers* and concluded that women are faster than men. As they write, “the gap between men and women shrinks as running distance increases” (Le Mat et al., 2023, p. 217), and “female ultra runners are faster than male ultra runners at distances over 195 miles.” Although these authors’ analyses appear to be well done, a comparison of men and women finishers cannot provide reliable information about the performance sex gap because men and women *finishers* may not be representative samples of men and women *runners*, respectively. Their rationale also conflicts with the finding that ultramarathon records are faster for men than for women (Table 1).

**TABLE 1:** World records for ultramarathon events and the corresponding sex gaps.^a^ ^,^ ^b^

Event	Finish time		
Race distance	Men	Women	% Faster
50 km	2:38:43	2:59:54	13.4
50 mi	4:48:21	5:40:18	18.0
100 km	6:05:35	6:33:11	7.5
100 mi	10:51:39	12:42:40	17.0
Distance (km)			
Race Duration	Men	Women	% Faster^c^
6 h	98.496	85.490	15.2
12 h	177.410	153.600	15.5
24 h	319.614	270.116	18.3
48 h	473.495	435.336	8.8
6 days	1036.800	883.631	17.3

^a^ Records ratified by the International Association of Ultrarunners https://iau-ultramarathon.org/iau-records.html Accessed on: 28 January 2024.

^b^ More information about ultramarathon sex differences is given by Senefeld et al. (2016).

^c^ For a given distance, percent farther equates to percent faster. For instance, for the 6-day event, the men’s record is 17% farther than the women’s record, which also means that the average speed for the men’s record is 17% faster than the average speed for the women’s record.

Finally, some claims of women dominance at long distances are made by researchers who omitted the relevant contradictory evidence. Bam et al. (1997) argued that the men-women gap *decreases* as race distance *increases* but did not mention that the world records show no such pattern. In fact, the men-women record gap has long equaled about 10% in every Olympic running event (e.g., Hallam and Amorim, 2022), and the sex gap is *greater* at most ultramarathon distances (Table 1). Bam et al. and Beneke et al. (2005) listed physiological sex differences that purportedly favor women distance runners, including smaller body size, greater resistance to pain, and greater oxidation of ingested glycogen, yet they cited none of the oft-cited sex differences that favor male runners, such as longer limbs, greater muscle mass, less percentage body fat, and higher VO_2_ max (e.g., Geary, 1998, p. 213; Cheuvront et al., 2005; Seiler et al., 2007; Tiller et al., 2021; Hunter et al., 2023, p. 213). In fact, a recent panel of experts concluded that men outperform women in athletic events requiring endurance, muscle strength, speed, and power because of “fundamental sex differences dictated by their sex chromosomes and sex hormones at puberty, in particular, testosterone” (Hunter et al., 2023, p. 2328).

## Researcher bias

Some of the researcher behaviors described above might be due to a poor understanding of data or sport, but most are better explained by researcher bias. For instance, the blatant cherry picking of performance data and the selective reporting of physiological sex differences recounted above are not easily attributed to naivete, especially when done by highly educated scientific researchers. Moreover, the deceptive research practices that underlay the claims of reversing sex gaps always worked in *favor* of the authors’ claim—never *against* it, which is evidence of systematic bias (Jussim and Honeycutt, 2023). Simply put, the world records indisputably demonstrate that the gap between men and women world records has remained large since the 1980s, and yet some researchers chose to ignore these and other relevant data and instead argued that elite women will dominate elite men.

Inappropriate research practices are certainly not limited to the field of exercise physiology. In recent years, methodologists have concluded that unplanned analyses of data and other researcher behaviors known as *p*-hacking are largely responsible for the replicability crisis in the biological and social sciences (e.g., Wicherts, 2017; Nelson et al., 2018; Bishop, 2019). Several remedies have been proposed, though most of these remedies are better suited for proposed experiments rather than for the kinds of retrospective studies described in this piece. For example, scientific journals could require that submitting authors preregister their planned data analyses so that they cannot repeatedly conduct *post hoc* analyses until they obtain the desired result (Wicherts, 2017; Nelson et al., 2018).

The unfounded claims of reversing men-women record gaps have almost certainly influenced popular beliefs. The projections in *Nature* by Whipp and Ward (1992) and Tatem et al. (2004) received worldwide coverage in the news media, and more recent claims of women dominance of ultramarathons have been disseminated many times in the popular press. For instance, numerous news stories have repeated the claim that women outrun men at very long distances because of physiological advantages (e.g., Brueck, 2020; Guiberteau, 2024). This in turn bolsters the misconception that the performance gap between men and women athletes is small, disappearing, and due to social influences rather than physiology.

The deceptive research behaviors that underlie the claims of reversing sex gaps in running performance might seem benign because predictions of athletic performance have little practical relevance, but any misleading scientific claim can be harmful. Bias or deception by scientists in any discipline justifiably reinforces the public’s distrust of science, and unfounded claims of reversing men-women athletic record gaps might influence people’s views about public policy. For instance, people who believe that the best female athletes can compete with (or outperform) the best males might be less likely to support the public financing of girls’ sports. Why support both boys’ and girls’ teams if girls can compete with boys? Or people might underestimate the impact of permitting post-pubescent biological males who identify as females participate in female-only athletic competitions, which is permitted in the United States in many high schools^5^ and the governing body of university sports.^6^ To be sure, these kinds of policy decisions are ethical questions and thus not answered by science, but people can better answer such questions when scientists faithfully characterize the evidence.

## Footnotes

1 The mean men-women record gap in Olympic events equaled 10.2% in January 1990 and 11.2% in January 2024. This comparison excludes women marathon records aided by men pacers; if these records are included, the 2024 sex gap equals 10.7%. The analysis also excludes the 400-m hurdles and the 3000-m steeplechase because the barriers in these events are 15.2 cm (6 in.) higher for men than for women, which complicates comparisons of men and women performances.

2
http://age-records.125mb.com/

3 Badwater 135 ultramarathon https://www.badwater.com/results-history/

4 Although both Whipp and Ward (1992) and Tatem et al. (2004) assumed that world records improve at a linear rate, Whipp and Ward expressed the records in units of *mean speed* (and assumed a linear increase), whereas Tatem et al. expressed the records as *finish times* (and assumed a linear decrease). It is mathematically impossible for both mean speed and finish time to improve linearly over time because the two measures vary inversely (distance = rate x time). Thus, the projections by Whipp and Ward and Tatem et al. contradict each other.

5
https://www.espn.com/espn/story/_/id/38209262/transgender-athlete-laws-state-legislation-science

6
https://www.ncaa.org/sports/2022/1/27/transgender-participation-policy.aspx
